# Automated Keratoconus Detection by 3D Corneal Images Reconstruction

**DOI:** 10.3390/s21072326

**Published:** 2021-03-26

**Authors:** Hanan A. Hosni Mahmoud, Hanan Abdullah Mengash

**Affiliations:** 1 Department of Computer Sciences, College of Computer and Information Sciences, Princess Nourah bint Abdulrahman University, Riyadh 11351, Saudi Arabia; HAhosni@pnu.edu.sa; 2 Department of Information Systems, College of Computer and Information Sciences, Princess Nourah bint Abdulrahman University, Riyadh 11351, Saudi Arabia

**Keywords:** keratoconus detection, 3D eye construction, cornea, depth calculation, machine learning

## Abstract

This paper presents a technique for the detection of keratoconus via the construction of a 3D eye images from 2D frontal and lateral eye images. Keratoconus is a disease that affects the cornea. Normal case eyes have a round-shaped cornea, while patients who suffer from keratoconus have a cone-shaped cornea. Early diagnosis can decrease the risk of eyesight loss. Our aim is to create a method of fully automated keratoconus detection using digital-camera frontal and lateral eye images. The presented technique accurately determines case severity. Geometric features are extracted from 2D images to estimate depth information used to build 3D images of the cornea. The proposed methodology is easy to implement and time-efficient. 2D images of the eyes (frontal and lateral) are used as input, and 3D images from which the curvature of the cornea can be detected are produced as output. Our method involves two main steps: feature extraction and depth calculation. Machine learning from the 3D images dataset Dataverse, specifically taken by the Cornea/Anterior Segment OCT SS-1000 (CASIA), was performed. Results show that the method diagnosed the four stages of keratoconus (severe, moderate, mild, and normal) with an accuracy of 97.8%, as compared to manual diagnosis done by medical experts.

## 1. Introduction

Keratoconus describes non-inflammatory disease usually depicted by progressive thinning, which cause cornea deformation. Affected patients will incur some extent of distorted vision and photophobia (light sensitivity) [1,2]. Keratoconus causes corneal cone shaping deformation, which leads to abnormalities, such as diverging of light on the retina that might lead to loss of vision. Recently, it has been noted that an increased number of people are being diagnosed with keratoconus [1]. Keratoconus affects 1 in 1500 individuals as prevailing from many epitomic reports [1,2,3].

With this high occurrence percentage needing medical attention in detection and timely treatment, automated rapid detection has become necessary.

Keratoconus is detected clinically using many signs, such as the Vogt striae and Fleischer signs. Keratoconus patients also display Munson and scissoring reflex signs. Medical examinations are usually done in a clinical setting using slit lamp examination techniques [1,2]. Imaging tools that implement Scheimpflug and corneal topography are also usually used. All of these examination methods are expensive and are performed by only experienced optometrists. Automated rapid detection has become necessary due to the increased number of keratoconus sufferers, as it is estimated that 1 in 1500 people in the population are affected.

Some computerized techniques have been proposed to detect keratoconus using corneal topography maps. These methods utilize machine learning, deep learning, and neural networks. Most perform keratoconus detection by using topography maps of both eyes [2,3].

Other approaches of 3-D Cornea construction from Microscopy Images have been introduced [4]. In Reference [5], the authors introduced automated measurement of corneal haze in optical tomography images. In addition, feature fusion techniques have been utilized for Keratoconus diagnosis 2-D photographed images [6].

In this paper, we propose an automated detection of Keratoconus by constructing 3-D images from the frontal and lateral 2-D images of the eye.

The rest of this paper is organized as follows. The background and literature survey are presented in Section 2. Section 3 presents the proposed features-extraction algorithm, depth calculation, and 3D corneal image construction. It also delineates the proposed keratoconus detection method. Results are discussed in Section 4 by comparing existing methods. Finally, the paper concludes in Section 5.

## 2. Background and Literature Survey

An automated keratoconus detection method can provide a solution for needed medical attention and timely treatment for Keratoconus diagnosis. Digital image processing can provide a solution for automated keratoconus detection, especially in rural areas. In Reference [6], Daud et al. devised a keratoconus detection method via digital image analysis processing. They tested their method on 140 cases captured by a smartphone, and achieved an average accuracy of 96.03%. Ali et al., in Reference [7], developed a technique that utilizes specific details from topographic maps. They used image analysis and processing to detect keratoconus in a computerized manner, identifying 12 features from the topographic maps. They collected data from 40 patients, and attained an accuracy of 90%. In Reference [8], the authors proposed a depth optimization method for 2D-to-3D conversion based on RGB-D images. These measurements can aid a great deal in keratoconus correction procedures. In Reference [9], the authors presented an affordable method of keratoconus detection using a smartphone. Experimental results of their proposed method show detection of severe and moderate keratoconus cases with accuracies of 93% and 67%, respectively. The authors in Reference [10] present a depth optimization technique for 2D-to-3D reconstruction based on RGB images that can be used in ocular disease detection.

Table 1 compares automated keratoconus detection methods according to the methodology used, the dataset, and the accuracy attained in experimental results.

3D eye-image construction is of great importance in the field of eye disease detection. 3D imagery has had a major impact in fields such as lateral disease detection and machine vision. 3D images incorporate depth information that can aid in different applications. For example, depth information can help diagnose many diseases that require depth calculations such as brain tumors and Cornea diseases [11,12]. However, 3D images are not always available. Thus, 3D image construction is usually utilized for such applications.

Many researchers have introduced techniques to reproduce 3D images from 2D images.

The authors in Reference [11] presented 3D edge reconstruction from 2D images utilizing a correlation algorithm with very high accuracy. The authors in Reference [12] recently proposed a novel 3D imaging approach for reconstructing 3D surfaces of a moving particle by recording its rolling motion through a digital microscope. The project discussed in Reference [13] constructed 3D images from 2D X-ray images of patients’ bones. They generated 3D images utilizing a machine learning method that is independent of the X-ray imaging setup. The authors in Reference [14] achieved depth-finding in real time utilizing statistical-based learning techniques, concluding that accuracy grew with an increased number of images in the training set. In Reference [15], Scarpa et al. obtained a 3D cornea reconstruction utilizing image sequences from a confocal microscope. They proved that it is possible to examine the cornea as a 3D complex and obtain imaging along the *x*, *y,* and *z*-axes. Table 2 describes some 3D image construction techniques that can aid in corneal disease detection.

The contributions of our proposed paper are explained below.

**Figure 1:**
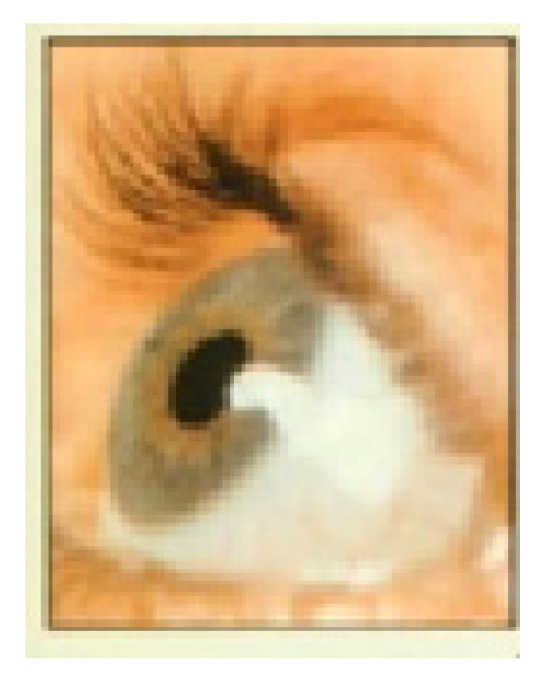
Normal eye.

**Figure 2:**
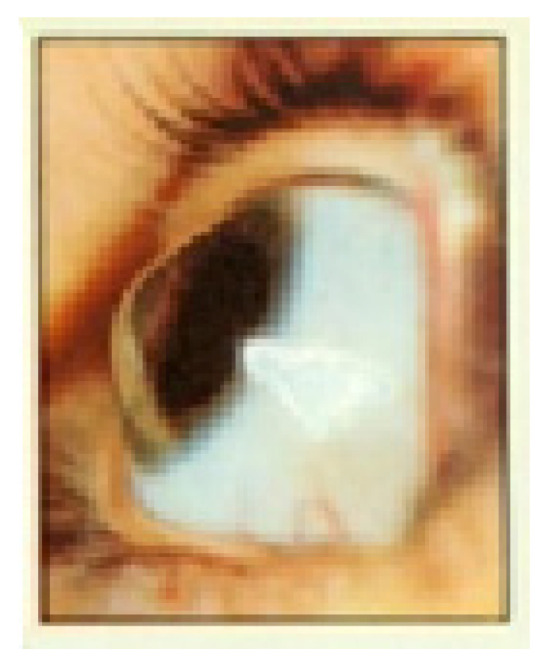
Keratoconus eye.

We emphasize on the 3D construction of corneal images to diagnose keratoconus and its stages by computing the curvature inclination angle of the cornea. Images of the structure of the eye, both normal and affected by keratoconus, are shown in Figure 1 and Figure 2. A lateral view of two images, a normal eye and an eye that has keratoconus, is seen. It is apparent from Figure 2 that the affected cornea forms a cone-shaped figure (Images used with permission [16,17]).

The presented method proposes the utilization of only two images of the eye, frontal and lateral, to efficiently generate a 3D image of the cornea. We utilized the Dataverse dataset found in Reference [16] for training. In total, 450 corneal images (250 keratoconus and 200 normal), including Ectasia Screening Index (ESI) keratoconus indices, curvature angle, and elevation, are provided by the dataset.

The second contribution is the utilization of scale-invariant feature transform (SIFT) features, which are invariant to the scale and orientation of the images. They are also invariant to illumination differences and robust to noise. SIFT can make it feasible to match inputs against other images with local features. This aids the training phase to a large extent. It has to be noted that Gaussian multivariate distribution is utilized to compute in-depth information.

The third contribution is that the proposed technique can achieve rotation of the cornea at any side view angle. This feature can help in the measurement of the stage of keratoconus and can compute the angle of curvature efficiently.

**Figure 3:**
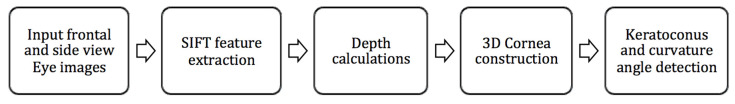
Block diagram of the proposed method.

The block diagram of the structure of the proposed technique is presented in Figure 3.

In the first step, two 2D frontal view and side view images of the eye are captured and used as input. Then, SIFT feature extraction is employed on the input images to identify image landmarks from local geometric deformations in multi-oriented and multi-scaled planes. Depth information is then extracted from the lateral view image of the eye to determine the points of interest. A 3D reconstruction of the cornea is built from two 2D segmented images imaged in orthogonal directions (frontal and lateral). Then, the training stage using the proposed Convolutional Neural Network (CNN), which takes 3D images as input and produce keratoconus detection. Detailed description of the steps in the block diagram are depicted in the following subsections.

## 3. The Proposed Method

In this section, we describe the proposed method in detail. The complete technique starts with cornea detection and feature extraction from 2D images, which is followed by an in-depth calculations’ algorithm leading to the reconstruction of the 3D image.

### 3.1. Cornea Detection and Features Extraction

In the first step, two 2D images of each eye are captured as input: a frontal view and one side view. The images are then scanned to detect the cornea and encompass specific marks on the eye region. Frontal and side view corneal images from the Harvard Dataverse database [16] were utilized for experimental purposes. The method uses circular Hough transform to detect the iris from the frontal image [17,18].

Hough transform can detect a circular Iris shape in the eye image.

For a circle with radius r, the characteristic equation of a circle at center (m, n) is given by:

(1) $${\left(x-m\right)}^{2}+\;{\left(y-n\right)}^{2\;}={\;r}^{2}$$

This circle is described by Equations (2) and (3).

(2) $$x=m+r\cos\left(\propto \right)$$

(3) $$y=n+r\cos\left(\propto \right)$$

where $x\cos\left(\propto \right)+y\sin\left(\propto \right)=r$

Hough transform searches for the parameters (m, n, r), which leads to the points *x_i_* and *y_i_*. Those points lie on the circumference of the required circle.

The center and radius of the iris are computed from the detected iris circle. These features will be utilized in the side view image of the eye to compute landmarks on the iris area, which will then be used to generate a 3D image of the eye. Input images pass through a preprocessing phase that detects the eye area to be used for further processing. Multiple 2D images can be utilized to recover 3D information by cracking a pixel-wise correspondence that can be extracted from the SIFT algorithm on two frontal and lateral views of the cornea.

### 3.2. Features Extraction and Depth Calculation

#### 3.2.1. Features Extraction

Finding distinct features of the eye area is crucial for detecting the iris and its dimensions. To this end, we employ the infamous scale-invariant feature transform [17,18]. SIFT has been used in feature selection in corneal images [19]. Keratoconus detection using curvature-based topography and SIFT feature extraction are well-used methodologies [20,21,22].

Other feature extraction methods, comparable to SIFT, are Speeded Up Robust Features (SURF) and Oriented FAST and Rotated BRIEF (ORB) [23,24]. The computational cost of ORB is lower than SIFT and SURF [25]. However, the problem with ORB is that it produces huge quantity of features, which leads to an enlarged time for matching and will increase the overall image matching computational cost. In the comparison results published in Reference [26], in image matching applications, SIFT and SURF are the best in scale invariant feature indicators on the foundation of scale variations. On the other hand, ORB is the least scale invariant. However, other variations of ORB, namely ORB (1000), is the best with respect to the rotation invariant. ORB is the best affine invariant as compared to the other algorithms. The algorithm SIFT has better accuracy for rotational variations. Although ORB is very efficient in detecting a very large number of features, it was found that it requires prolonged matching time. The huge number of features increases the total image matching computational time with respect to SIFT [24].

**Figure 4:**
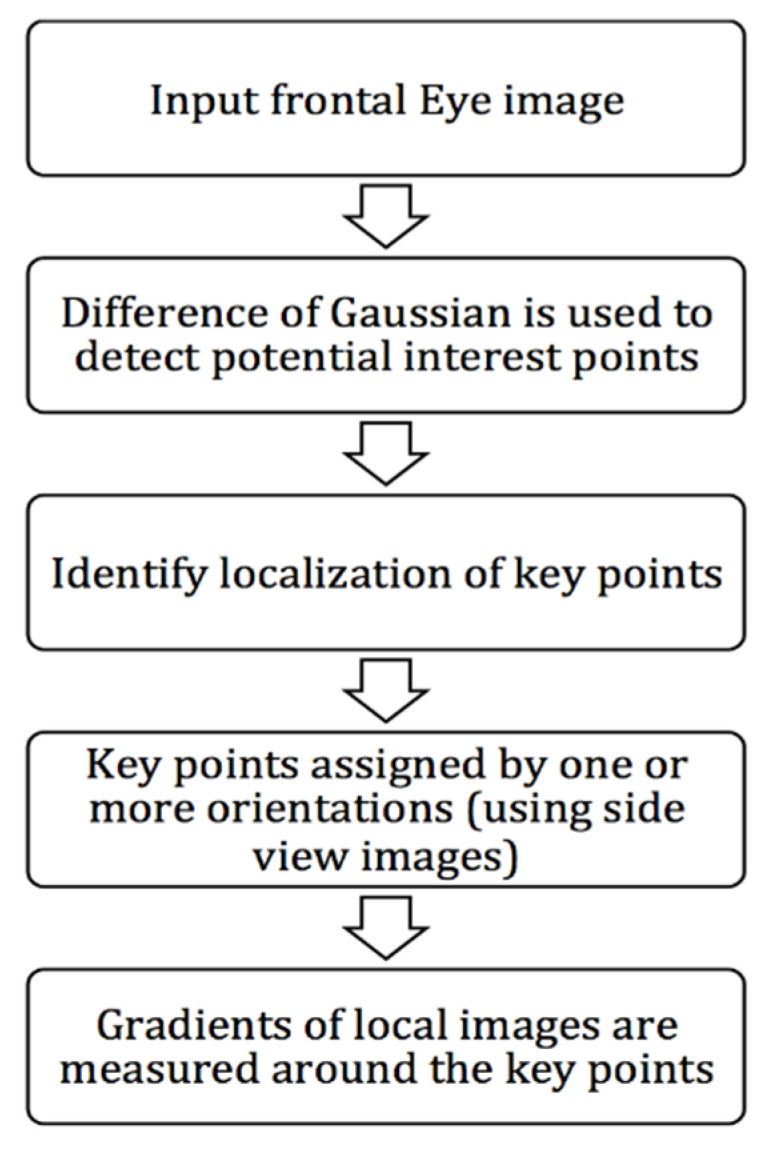
The stages of the feature extraction method.

Local features are identified through a staged filtering technique detecting the points in scale space that are stable and identifying image landmarks from local geometric deformations in multi-oriented and multi-scaled planes. Reconstructing the 3D shape of the eye relies heavily on these features. The stages of the feature extraction method are described in Figure 4.

The SIFT technique identifies locally distributed extreme points by relating pixel points in the Gaussian space with adjacent points in the domain region. The extreme points establish all feature points.

Key points of the two images are identified by finding the nearest neighbor-points. It is often the case that the second match is very close to the first. It can happen because of noise or, in such a case, the ratio between the first and second closest-distance is computed and considered.

**Figure 5:**
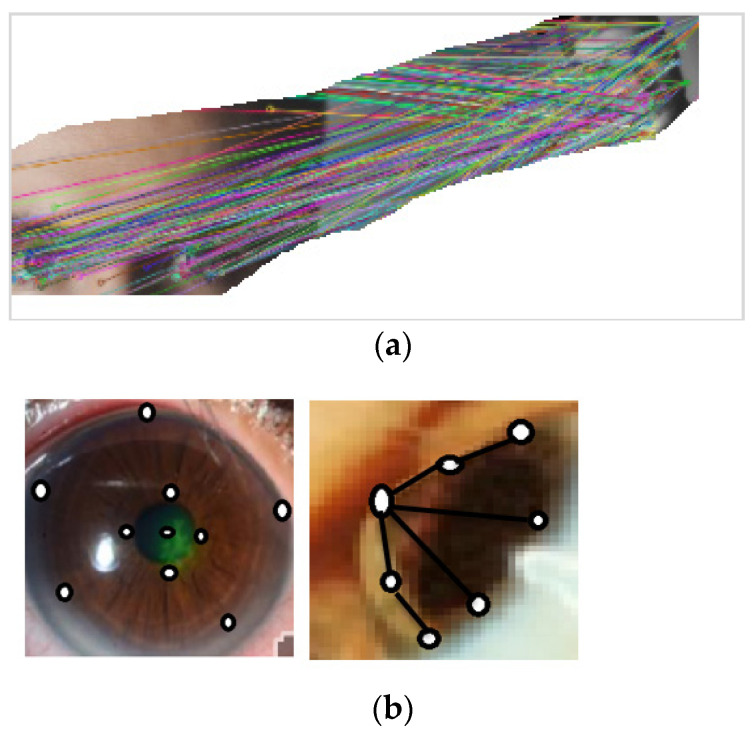
(**a**) Example of the output of the scale-invariant feature transform (SIFT) algorithm. (**b**) Dense features extraction.

This approach is effective in producing several dense features. Figure 5a presents the output of the implementation of the SIFT algorithm between frontal and side views of an eye image where key point features are matched between the two views. Figure 5b presents outputs of the SIFT algorithm in side view images of normal and affected eyes. Black circles show the positions of feature points of the eyes and iris regions. Black lines are added by the algorithm to convey depth information, which can be calculated to aid in the 3D construction of the eye.

#### 3.2.2. Depth Calculation

**Figure 6:**
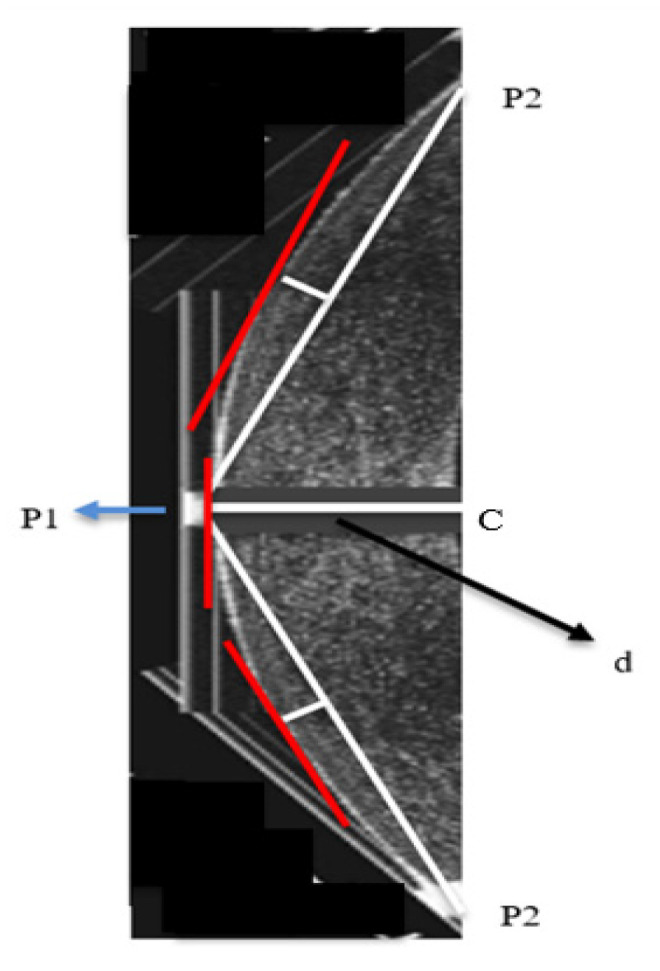
Lateral view of the cornea.

Depth information is collected from the lateral view image of the eye, as illustrated in Figure 6.

Hough transform will detect the curvature of the cornea from the frontal view and will be aligned to the side view, where points on the circumference of the cornea will be detected. Tangents to the rightest point are drawn. The highest point on the curve and the lowest points, namely p1, p2, p3, are determined. From this point, a right-angled triangle is drawn, where p1 is the vertex of the triangle and p2 is the other vertex. The length of the side d is calculated and it is the depth of the cornea from its highest point.

The steps are as follows:

1. Determine points p1, p2, and p3 using Hough transform
2. Calculate C: the mid-point between p2 and p3
3. Calculate d using the Pythagoras theorem

### 3.3. Reconstruction of the 3D Image

After calculating depth information, 3D data of the cornea regions are now available. A 3D reconstruction of the cornea is built from two 2D segmented images imaged in orthogonal directions (frontal and lateral). An orthogonal plane can be fused without assuming prior knowledge. The intersections of the images in the orthogonal planes can be used to recognize alignment reference points, using homogenous translational transform T by factors x, y, and z. Points in the orthogonal images in 2D can be converted into 3D points in the 3D plane using Equations (4) and (5).

The local points in the frontal image, the local point in the lateral orthogonal plane, and the global corresponding point in the 3D plane are, respectively:

(4) $$\left[\begin{array}{c} x1\\ y1\\ 0\\ 1 \end{array}\right],\;\left[\begin{array}{c} x2\\ y2\\ 0\\ 1 \end{array}\right]\mathrm{and}\;\left[\begin{array}{c} x3\\ y3\\ z3\\ 1 \end{array}\right]\;\mathrm{Where},\;\left[\begin{array}{c} x3\\ y3\\ z3\\ 1 \end{array}\right]=\;\left[T\right]*\left[\begin{array}{c} x1\\ y1\\ 0\\ 1 \end{array}\right]\;\mathrm{and}\;\left[\begin{array}{c} x3\\ y3\\ z3\\ 1 \end{array}\right]=\;\left[T\right]*\left[\begin{array}{c} x2\\ y2\\ 0\\ 1 \end{array}\right]$$

(5) $$\left[\begin{array}{c} x1\\ y1\\ 0\\ 1 \end{array}\right]=\;\left[\begin{array}{c} 0\;\;\;\;0\;\;\;\;\;1\;\;\;\;\;z3-x1\\ 0\;\;\;\;1\;\;\;\;0\;\;\;\;\;y3-y1\\ \;1\;\;\;\;0\;\;\;\;\;0\;\;\;\;\;\;x3-x2\\ 0\;\;\;\;0\;\;\;\;\;0\;\;\;\;\;\;\;\;\;\;\;\;\;\;\;1\;\; \end{array}\right]*\left[\begin{array}{c} x2\\ y2\\ 0\\ 1 \end{array}\right]$$

where x1, y1 and x2, y2 are the points in the 2D plane to be fused into the 3D point x3, y3, z3.

The problem in Equation (5) is formulated as an optimization problem to minimize the total Euclidean distance, in 3D space, between all of the corresponding points.

We tested our alignment procedure on data from the Dataverse dataset [16], which is available online. The data consist of 200 normal cornea cases and 250 keratoconus cases in which each have a frontal and lateral RGB image and a 3D image of the cornea. The testing procedure is to choose a patient case from the Dataverse dataset (normal or keratoconus), and to use the frontal and lateral images to generate a 3D reconstruction of the cornea. We established point-to-point correspondence with our proposed geometrical method. The objective of the testing was to minimize the sum of square differences of pixels between the processed 3D image and the Dataverse’s stored 3D image to establish a match. The error function is shown in Equation (6).

(6) $$\min_{\forall \left(\mathrm{stored}\right)}\left(E=\sum x,y,z\left(I_{\mathrm{construct}}\left(x,y,z\right)-I_{\mathrm{stored}}\left(x,y,z\right)\right){\;}^{2}\right)$$

where I*_construct_* (x, y, z) is the intensity level of the constructed image at point x, y, z and I*_stored_* (x, y, z) is the intensity level of the stored image at point x, y, z.

Evaluation of the construction of 3D corneal images from two frontal and lateral images is presented in Section 4.

### 3.4. Keratoconus Detection Algorithm

#### 3.4.1. Implementation of the Convolutional Neural Network (CNN)

There are few studies utilizing neural networks in keratoconus automated detection. Researchers in Reference [21] presented keratoconus detection methods using a neural network approach. Scheimpflug tomography in topographically normal patients and keratoconus cases was used as described in Reference [22].

In this section, we present our proposed CNN methodology for automated keratoconus detection. The keratoconus-detection algorithm, using 3D reconstructed corneal images, was implemented and tested utilizing a convolutional neural network. 3D RGB corneal images were the inputs to the proposed algorithm. This section presents the learning process related to the convolutional neural network.

The implemented CNN processes the 3D images as input data, utilizing weights on neurons’ inner connections. Continuous tuning of these weights in the learning process is performed to decrease an error in the classification process as well the learning process.

#### 3.4.2. The Proposed CNN for Keratoconus Classification from the 3D Constructed Cornea Images

The proposed CNN is used to extract features in an automated way. The CNN consists of four convolutional, four max pooling, and two fully connected layers. The kernels sizes are: 3 × 3 × 3 in the convolutional layers, and 2 × 2 × 2 in the pooling layers. The feature kernels are 96, 128, 256, and 512 in the convolutional layers, respectively. In the first convolutional layer, 3 × 3 × 3 ninety-six filters are applied to 227 × 227 × 227 input images. The max pooling layer applies 2 × 2 × 2 filters to reduce the size of the preceding convolutional layer output. The reduced images are handled by the following convolutional layers after applying the second and third pooling layer. The model applies the rest of the layers until finally reaching the two fully connected layers with all neurons connected to neurons of the previous fully connected layer. A SoftMax classifier is utilized to classify the 3D cornea images. The output of the last fully connected layer is fed to the Softmax classifiers as an input. The number of samples in each training cycle is set to 32 with a learning rate of 0.01.

**Figure 7:**
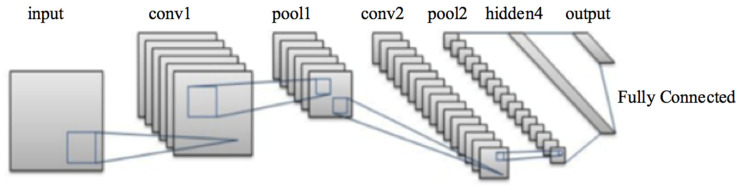
Architecture of the proposed convolutional neural network (CNN).

The architecture of the proposed CNN is demonstrated in Figure 7, and the description of the CNN layers is described in Table 3. The proposed CNN is trained in 30 epochs.

Feature extraction is performed through the convolution layer by using the 3D image of the cornea as an input and generating the image matrix as an output. The image matrix is a representation of the pixels’ relationship through machine learning techniques, which capture the characteristics of the images through filters.

In the 3D corneal images, corneal gray scale intensities are utilized. If the cornea is affected by keratoconus, the gray scale intensity levels exhibit a larger area and an elevation factor. Dark grey intensity levels specify high elevation. Light intensity levels specify a flat elevation level.

**Figure 8:**
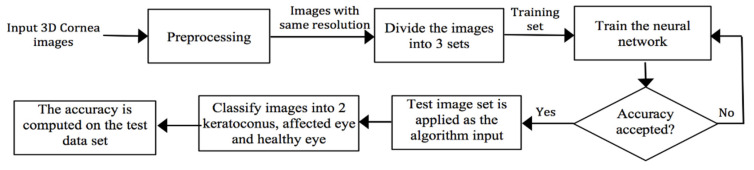
The implementation of the convolutional neural network (CNN).

Preprocessing of the input images is performed to obtain the same resolution. Images are then divided into three sets: the training set for the CNN, the validation set, and the testing set. The complete algorithm is depicted in Figure 8.

Preprocessing is a necessary step to normalize input images to a unique space. All images are normalized to a unique spatial space by resampling, which is followed by extraction of the ROI that encloses the cornea area. The 3D images are filtered utilizing 3D Gaussian filter to reduce high-frequency noise.

#### 3.4.3. Geometrical Features Extraction

**Figure 9:**
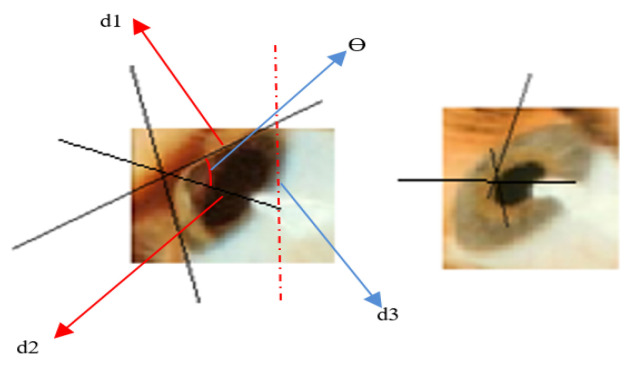
Corneal curvature slopes and angles computation.

The proposed method utilizes geometric measurements to compute corneal curvature slopes and angles. Curved, steep areas are characterized by an increased slope (with respect to the vertical axis), while flat corneal areas are of much lesser slope, as shown in Figure 9.

After classifying the image as keratoconus-affected, we use the feature extraction algorithm to identify the stage of keratoconus.

The slopes are normalized according to the relative scale of the cornea-enclosed rectangle and give a perspective of the corneal curvature. The method performs classification of the 3D corneal images, according to the slope of the curvature in three dimensions. This enables the identification of keratoconus stages.

There are three stages that characterize keratoconus. These stages are detected through geometrical metrics of the outer shape of the affected cornea, or by measuring its thickness. In our research, we used the geometry of the outer shape of the cornea by measuring the steepness of the greatest corneal curvature from the reconstructed 3D corneal images.

The angle of curvature = 1800− θ, where θ is calculated from the three displacements d1, d2, and d3, as in Equation (7).

(7) $$\theta =\cos^{-1}\left(\left(d22+d32-d12\right)/\left(2\times d2\times d3\right)\right)$$

The steepness of the greatest curvature is measured as angles of the slope of straight lines drawn adjacent to the cornea. If the degree of steepness is less than 45°, the instance is diagnosed as a mild case of keratoconus. From 45° to 52°, it is classified as an advanced case. If greater than 52°, it is a severe case.

## 4. Experimental Results

### 4.1. Simulation Results for Computing the Angle of Curvature of Keratoconus

In this subsection, we present simulation results for computing the angle of curvature of keratoconus by computing the correlation between the actual angle of curvature of keratoconus in patients from a physician’s diagnosis, and the angle of curvature extracted from 3D corneal images of the patients.

**Figure 10:**
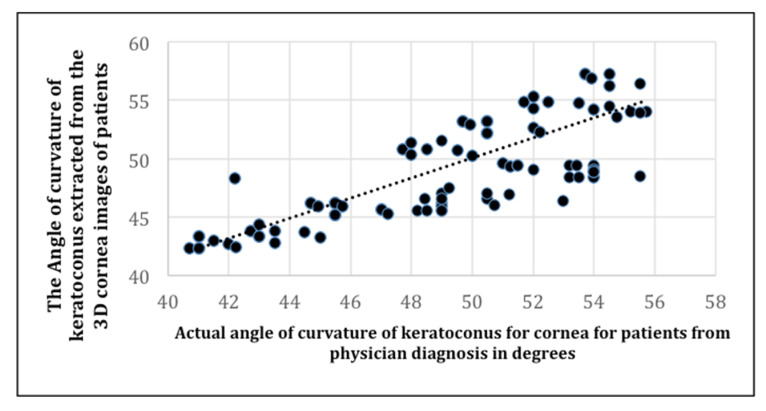
The correlation between the actual angle of curvature of keratoconus in patients, and the angle of curvature of keratoconus extracted from 3D corneal images of the patients.

Figure 10 is a presentation of the simulation results of the correlation between the actual angle of curvature of keratoconus in patients with an actual physician’s diagnosis, and the angle of curvature of keratoconus extracted from the 3D corneal images of the patients.

**Figure 11:**
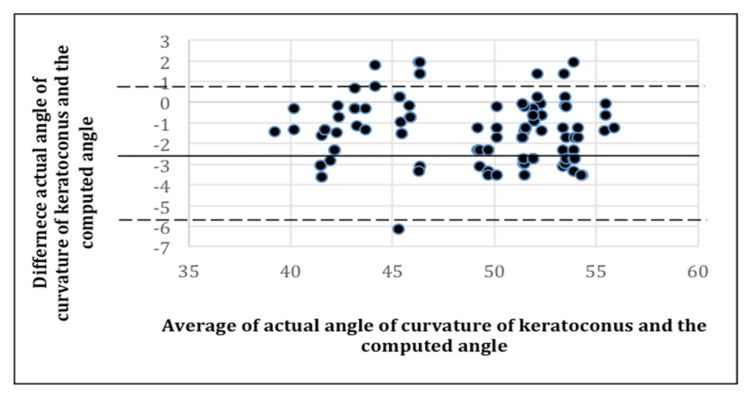
Bland-Altman plot.

Figure 11 is a presentation of the Bland-Altman plot of the actual angle of curvature of keratoconus in patients from an actual physician diagnosis versus the angle of curvature of keratoconus extracted from the 3D corneal images of the patients.

The correlation plot in Figure 10 depicts the positive correlation between the actual angle of curvature diagnosed by a physician, and the angle of curvature measured by our algorithm. The experiment shows a strong linear relationship between the physician’s diagnosis and our predicted detection. 

A strong positive correlation using Pearson’s *r* (Equation (8)) is indicated, as *r* is greater than 0 and approaches 1.

(8) $$r=\frac{1}{n-1}\;\overset{n}{\underset{i=1}{\sum }}\left(\frac{x_{i}-\bar{x}}{{\mathrm{SD}}_{x}}\right)\;\left(\frac{y_{i}-\bar{y}}{{\mathrm{SD}}_{y}}\right)$$

We computed *r* in Equation (7) to be 0.95, which implies a high positive rate between the actual angle of curvature of keratoconus in patients, and the angle of curvature of keratoconus predicted by our algorithm using the data in Figure 10.

It should be noted that the Bland-Altman metric depicts the agreement of two measurements. The Bland-Altman metric is usually used in comparing a predicted computerized medical diagnosis (P) with actual diagnosis by medical personnel (A). The two measurements are the predicted angle of curvature and the actual angle of curvature of the keratoconus. The difference between P and A is where the upper and lower dotted lines denote 1.96 SD, which is the 95% limit of agreement. The difference between P and A is plotted against the mean of the manual P and A. The Bland-Altman metric between A and P indicates that the trend of the values of the two tests are of high similarity.

### 4.2. CNN Training Process Accuracy and Error

The proposed CNN has four convolutional and pooling layers with completely full connected layers, and a SoftMax layer. The proposed algorithm measures the slope of corneal curvature geometrically and calculates the angle of curvature.

*Results:* We extracted the medical diagnoses done by a physician for 250 cases. Medical evaluations were found fully in the dataset Dataverse.

Test validation was performed utilizing k-Fold cross validation and hold out testing.

**K-Fold Cross-Validation:**

The performance of the proposed CNN is measured using a k-fold cross-validation. The data is distributed into k partitions of nearly equal sizes. CNN training as well as validation are executed in k iterations. Each fold is employed for testing per iteration, while k-1 folds are employed for training. The model accuracy is computed as the average accuracy over all iterations. The results of the first experiments of the CNN utilizing 3D Cornea image results are depicted in Table 4.

The results show an enhanced accuracy in the 3D Cornea images, especially in the 10-fold. We separated the data into 10 equal folds. Each fold contains cases from each Keratoconus cases (normal, severe, moderate, and mild). Experiments established that k-fold testing with k = 10 has the higher accuracy. The cross-validation testing for different k is depicted in Table 4 for 3D constructed Cornea images.

For the 10-fold validation, the classification has an average of 2.2% error approximately, which is considered a very low classification error rate, especially when mild cases are included.

The confusion matrix for k = 10 is illustrated in Table 5. The database includes 58 images of mild cases, and 70 images of moderate cases. It contains 40 3D Cornea images of severe cases. Another 100 of normal 3D Cornea images are included.

The confusion matrix for k = 10 is illustrated in Table 5, where comparisons of the results of actual medical diagnoses with our proposed method is depicted. Our CNN demonstrates the ability to predict correct diagnoses of the four stages of keratoconus (severe, moderate, and mild) as well as normal, keratoconus-free cases, as indicated in Table 5 and Table 6.

The classifier is evaluated according to accuracy, sensitivity, and specificity, as defined in Equations (9)–(11) respectively.

(9) $$\mathrm{Accuracy}=\frac{\mathrm{TP}+\mathrm{TN}}{\mathrm{TP}+\mathrm{FP}+\mathrm{FN}+\mathrm{TN}}$$

(10) $$\mathrm{Sensitivity}=\;\frac{\mathrm{TP}}{\mathrm{TP}+\mathrm{FP}}$$

(11) $$\mathrm{Specificity}=\;\frac{\mathrm{TN}}{\mathrm{TN}+\mathrm{FN}}$$

where TP indicates true positive cases, TN indicates true negative cases, FP indicates a false positive detection, and FN indicates false negative cases.

The accuracy is defined as the percentage of correctly predicted cases in the test set, the sensitivity is defined as true positives rate, and the specificity is defined as a true negatives rate. These measures are computed and illustrated in Table 6.

Table 7 illustrates the comparison of results of The CNN for Keratoconus classification in the literature and our proposed CNN using k-Fold Cross-Validation, k = 10: training set (80%) and testing set (20%).

## 5. Conclusions

This paper presents an automated technique that reconstructs 3D corneal images from two 2D frontal and lateral corneal digital images. The 3D reconstructed images are used to detect keratoconus in an automated way. The proposed technique consists of four main steps: cornea detection in the 2D images of the eye and features extraction, features extraction and depth calculation, construction of the corneal 3D images, and automated detection of keratoconus and determination of its severity using the angle of curvature of the 3D reconstructed corneal images. Our experimental results show that the proposed method identifies keratoconus and its stages efficiently. The proposed method was validated by performing experiments and making comparisons with manual methods performed by professional medical experts, which are presumed to be ground truth. Quantitative results are compared with ground truth to prove the accuracy of this method.

By examining its accuracy, it can be seen that the proposed method can detect keratoconus comparably to the results of an actual medical diagnosis. The technique shows the ability to predict correct diagnoses of the four stages of keratoconus (severe, moderate, and mild) as well as normal keratoconus-free cases, with an accuracy of 97.8% in a total of 268 cases. These results are shown to be better than those achieved by the systems using image processing in References [6,9]. 3D reconstruction of the cornea can also be utilized to detect other diseases of the cornea and in educational paradigms.

For future work, we will concentrate on the computational cost of the proposed scheme. The complexity of the proposed scheme can leave many opportunities for enhancing speed-up of the individual steps. In this paper, we concentrated more on accuracy and precision rather than exploiting time complexity especially when the application does not have a real time requirement.

The proposed scheme can be utilized as an aid for ophthalmology personnel. Mass screening can be done using an automated system based on this scheme, since the specificity of the proposed method is relatively high at around 96%, which is the percentage of the true negatives that are correctly recognized by the proposed scheme. Therefore, the proposed system can identify a normal condition in an automated way and will not be needed for further investigation by medical experts. Ophthalmologists will concentrate on the positive cases that are detected by the proposed method.

## Figures and Tables

**Table 1:** Automated keratoconus detection methods.

Reference	Achievement	Method	Dataset	Results
[5] Dhaini, A.R.; et al., 2018	Corneal haze and demarcation line measurement.	Image analysis and machine learning	140 Keratoconus eyes for actual patients	The mean demarcation line is 295.9 ± 59.8 microns, and it is 314.5 ± 48.4 microns by medical personal.
[6] Daud, M.M.; et al., 2020	Keratoconus detection method	Digital image analysis processing	140 cases captured by smartphone	Accuracy of 96.03%
[7] Ali, A.H.; et al., 2018	Keratoconus supervised learning and detection	Support vector machine using image processing techniques	240 cases were attained from Al-Amal Eye Clinic in Baghdad utilizing a Pentacam	Accuracy of 90%
[9] Askarian, B.; et al., 2018	Diagnostic method for keratoconus detection	Usage of a smartphone	175 images of keratoconus cases	Accuracies of 93%, 67% in severe, and moderate cases, respectively.
The proposed method	Automated keratoconus detection	Depth calculation from 3D corneal image and machine learning	268 Corneal images of Keratoconus and normal cases	Accuracy is 97.8%

**Table 2:** 3D Image construction.

Reference	Achievement	Method	Dataset	Results
[11] Patoom. K., et al., 2019.	Reconstruction of 3D edge from 2D image	Correlation based algorithm	Images are a real object taken by a camera	Attains 100% accuracy
[12] Wang, S., et al., 2020.	3-D particle reconstruction	Utilizing multi-view 2-D motion and shape shading	3D camera images of moving wear particles	The performance undergoes degradation if occlusion occurs between particles
[13] Dixit, S., et al., 2019.	3D image-building from 2D X-Ray images	Conversion machine learning	Thirty samples of patient data (femur bones) have been acquired	Moderate accuracy with less processing time
[14] Jeni, A.L., et al., 2017.	Depth finding in real time	Statistical-based learning techniques	512 images of vertices	Accuracy increases by the number of images in the training set
[15] Scarpa, F., et al., 2007	3D Corneal image reconstruction	Fusion of confocal microscopy images	Images covering the whole corneal thickness in normal subjects with the Confoscan4 confocal microscope	Failed in 3% of images

**Table 3:** Summary of the proposed convolutional neural network (CNN) architecture.

Layer	Number of Kernels	Kernel Size	Output Size
Convolutional layer	96	3 × 3 × 3	96 × 114 × 114 × 114
Pooling layer		2 × 2 × 2	96 × 113 × 113 × 113
Convolutional layer	128	3 × 3 × 3	128 × 111 × 111 × 111
Pooling layer		2 × 2 × 2	128 × 56 × 56 × 56
Convolutional layer	256	3 × 3 × 3	256 × 50 × 50 × 50
Pooling layer		2 × 2 × 2	256 × 25 × 25 × 25
Convolutional layer	512	3 × 3 × 3	512 × 12 × 12 × 12
Pooling layer		2 × 2 × 2	512 × 6 × 6 × 6
FC			1000 × 1 × 1 × 1
FC			1000 × 1 × 1 × 1

**Table 4:** Cross-validation for keratoconus detection.

K-Fold Testing	Accuracy	Precision	Recall	F1-Score
K = 8	93.30%	92.71%	93.17%	92.83%
K = 10	97.8%	96.41%	97.23%	97.01%
K = 12	92.45%	91.80%	91.90%	90.86%
K = 14	88.98%	88.66%	88.72%	88.55%

**Table 5:** Keratoconus detection confusion matrix.

		Predicted Cases								
		Mild	Moderate	Severe	Normal	Total	TP	FN	TN	FP
**Actual Cases**	Mild	48	2	0	8	58	48	10		
	Moderate	2	66	2	0	70	70	0		
	Severe	0	0	40	0	40	40	0		
	Normal	2	2	0	96	100	96	4	96	4

**Table 6:** Accuracy, sensitivity, and specificity of the proposed system.

Metric	Computation
Accuracy	97.80%
Sensitivity	98.45%
Specificity	96.00%

**Table 7:** Comparison of results of the convolutional neural network (CNN) for Keratoconus classification and our proposed CNN, using k-Fold Cross-Validation, k = 10: training set (80%), testing set (20%).

Method	Accuracy	Sensitivity	Specificity
[5] Dhaini, A.R., et al., 2018	93.68%	94.60%	91.43%
[6] Daud, M.M., et al., 2020	96.03%	95.18%	94.44%
[7] Ali, A.H., et al., 2018	90.68%	92.10%	93.52%
[9] Askarian, B., et al., 2018	93.68%	94.60%	91.43%
The proposed methodology	97.80%	98.45%	96.00%

## Data Availability

Not applicable.
